# 3C3R Modified PBL Pediatric Teaching of Chinese Medical Students

**DOI:** 10.1371/journal.pone.0063412

**Published:** 2013-05-07

**Authors:** Haihong Xue, Jihong Qian, Lianwen Wang, Xiaojun Yuan, Yi Chen, Weilan Wu, Yan Chen, Kun Sun

**Affiliations:** Department of Pediatric, Xinhua Hospital, School of Medicine, Shanghai Jiaotong University, Shanghai, China; National Taiwan University Hospital, Taiwan

## Abstract

A Content, Context, Connection and Researching, Reasoning, Reflecting (3C3R) model is a conceptual framework for problem-based learning (PBL) problem design. We introduced the 3C3R-PBL method into a pediatric teaching plan, and evaluated its effectiveness and feasibility. The 3C3R model was applied in a pediatric problem design teaching plan “why the lips turn purple when a baby is crying”. All students were assigned either into a traditional PBL course or into a 9-step 3C3R model PBL course (3C3R-PBL). The performance outcomes of both groups were compared. For the PBL group, the proportion of students scoring ≥4 for content, context, and problem design connection, was 90.8%, 80.3%, and 64.5% respectively, while for tutors, it was 71.4%, 71.4%, and 28.6%; for researching, reasoning, and reflecting, the proportion of students scoring ≥4 was 81.6%, 55.3%, and 40.8%, while for tutors, it was 71.4%, 100%, and 57.1%. The learning difficulty was not considered high with only 31.6% of students and 42.9% of tutors rating the task as difficult. For the 3C3R-PBL group, the proportion of students scoring content, context, and connection, ≥4 was 100%, 98.4%, and 90.5%, while for tutors it was 100%, 100%, 83.3%; for researching, reasoning, and reflecting, the proportion of students scoring ≥4 was 95.2%, 88.9%, and 76.2%, while for tutors it was 100% for all 3 R components. Students and tutors were convinced by the content, case context, research process and reasoning process of both teaching plans, while scores for connection and reflecting were significantly improved when the PBL plan was amended by a 3C3R model (p<0.05) and the case learning difficulty was statistically increased (p<0.05). The 3C3R model, evaluated for the first time in China, was helpful for effective and reliable problem design in a pediatric PBL teaching plan for Chinese students.

## Introduction

**Figure 1:**
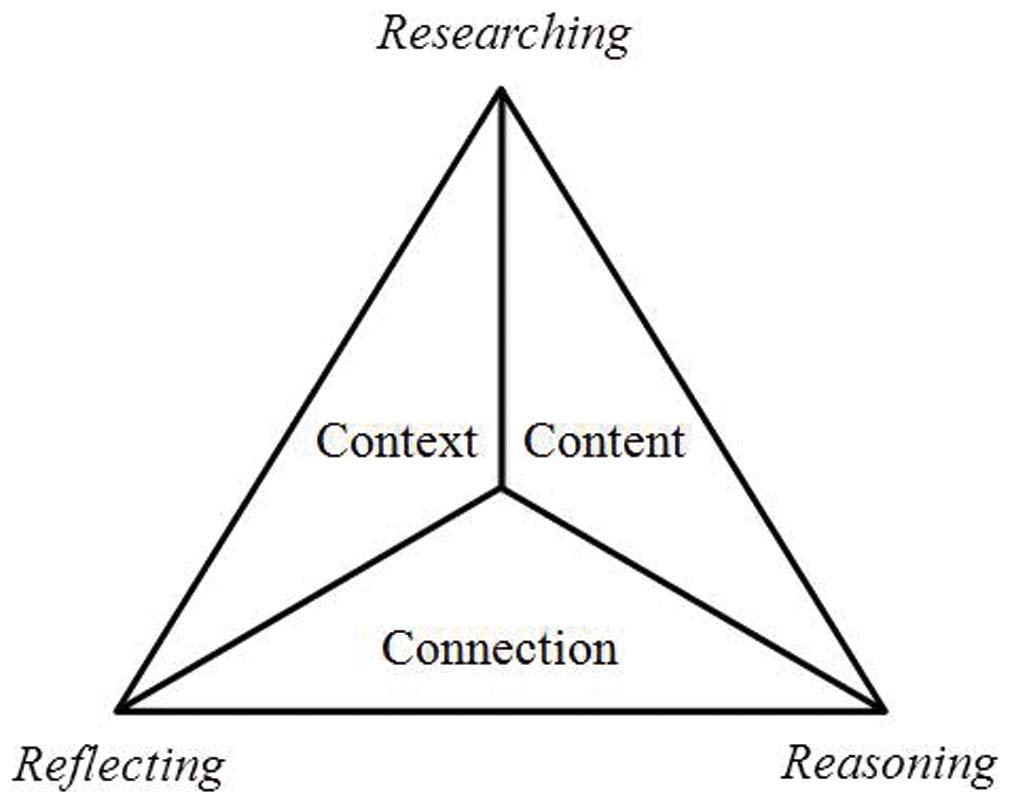
Framework of a 3C3R-PBL problem design.

Problem-based learning (PBL) is an innovative technique in the field of education whose purpose is to develop students' ability to apply knowledge to solve complex and realistic problems, as well as to assist students' in their development of advanced thinking and self-directed learning skills [1]. With the deepening of the PBL teaching practice in basic and higher education, the validity of PBL became a widely concerned point of discussion for a large number of scholars and practitioners and has been a topic of public debate since the late 50′s [2], [3], [4]. PBL advocates insist that PBL is more effective than traditional methods in reducing inert knowledge and in improving students' problem solving and self-directed learning abilities [5], [6]. Skeptics are adamant that PBL is expensive and inefficient, since it takes more time for students to achieve the desired learning outcomes compared to conventional teaching [7] and it is less effective than traditional methods, because its minimum guidelines do not meet the requirements of the human cognitive system [8]. In a PBL course, students work in groups and learning is achieved through solving a problem or series of problems rather than by passively learning from materials received from a tutor/instructor presented in a logical order and in accordance with a “textbook”. The content of a PBL course consists of addressing issues and information necessary to solve problems. The core of PBL therefore is a problem, making problem design the key to successful application of PBL [9], [10]. To address the challenging task of generating or selecting a problem, Hung developed a 3C3R model [11] to guide the design of effective, precise and reliable problems. Hung noted that if PBL problems are invalid, or if ambiguous information outside of the plan is contained in the issues, then the problem itself may lead to inappropriate (that is, insufficient, excessive or out of topic) content coverage, and thus to inappropriate problem-solving skills (above or below the learner's ability). Due to these shortcomings, invalid PBL problems will affect the group's ability to build upon previous knowledge and problem-solving processes, cause difficulties for students in raising learning problems covered in the original designed problems, affect students' self-directed learning, which will in turn affect the students in gaining the contents as well as affect their overall learning experience. Figure 1 shows the framework of a 3C3R model [11] applied in a PBL problem design, which is composed of two parts.

The core part of 3C3R contains 3Cs (**Content, Context and Connection)**, including the learning content and concept as a key point in a PBL problem design [11]. Three aspects for the students' knowledge, problem solving and self-directed learning capacity should be considered when designing the content and scope. In addition, the design should reflect the cognitive ability and PBL experiences of the students, so that suitable anticipated goals are developed. The 3R process (**Researching, Reasoning and Reflecting**), is closely related to and designed around the core of 3Cs. A 3R design focuses on mastering the necessary learning contents, developing the expected learning goals in appropriate depth, utilizing proper and effective research methods, using rational and efficient reasoning processes, while integrating conceptual knowledge processes and effective strategies to solve the problem [12]. Therefore, the problem of neglecting the PBL learning outcome due to too much emphasis on the depth of the learning content in the problem design can be avoided. 3R design can enhance the students' ability to solve problems in cognitive processes and can cultivate self-directed learning and study habits. In 2009, the 3C3R problem design model was further improved and a 9-step design process was developed to guide the problem design of PBL teaching, thereby increasing PBL effectiveness by analyzing and optimizing PBL problems [12]. Since its first implementation, PBL has become a prominent instructional method in medical and health science education throughout the world [13]; however, no uniform curricula and teaching material have been implemented for a pediatric PBL curriculum in China. Without uniform curricula, tutors must develop PBL curricula independently based on their different conditions [14], [15], [16]. By following the conceptual framework of a 3C3R problem design model, PBL curriculum designers will be able to systematically design more effective problems.

This study was designed to foster medical school students' abilities to master diagnosis, recognize clinical manifestations and complications, as well as determine the prognosis and learn the treatment principles of common congenital heart diseases (CHD) in children through discussions in a PBL teaching course based on their knowledge of Anatomy, Physiology, Pathology and Diagnostics. Although pediatric PBL problem design is well explored and established in China, to our knowledge this is the first time that a 3C3R-PBL model has been applied in China. Here we report our findings from comparing a PBL and a 3C3R-PBL model in a pediatric teaching setting and we discuss how a 3C3R-PBL model might be approved as a comprehensive, effective and feasible pediatric PBL teaching mode and concept in China.

## Subjects and Methods

### Subjects

Study subjects comprised 139 eight-year course, fifth grade students of the School of Medicine in the Shanghai Jiaotong University. 36 male and 40 female students, aged 22.35±0.68 years, attended the traditional PBL course (PBL group) and were randomly assigned into one of 7 subgroups; 30 male and 33 female students, aged 22.50±0.75 years attended the 9 step 3C3R model (3C3R-PBL group) and were randomly assigned into one of 6 subgroups. A tutor with at least 14 years pediatric clinical practice and teaching experience led each sub-group. Four tutors supervised both a PBL course and a 3C3R-PBL group as well (Table 1).

**Table 1:** Information about the tutor.

Group	Sex	Age	Teaching years	Degree
Traditional PBL+3C3R-PBL	Male	39	16	MD, Ph.D
	Female	45	22	MD, Ph.D
	Female	48	24	MD
	Male	41	17	MD
Traditional PBL	Female	40	17	MD, Ph.D
	Female	39	16	MD
	Male	40	14	MD
3C3R-PBL	Female	42	18	MD
	Male	38	15	MD, Ph.D

### The concrete method for PBL implementation

The core of PBL is the problem chosen, whose quality affects not only the performance of the group but also affects the students' autonomous learning and their interests in the learning content. Groups began their course with reasonable problems that were raised by their tutors and the students were tasked with solving the chosen problem. Students formed their personal views through reading literatures in related books or Internet and from discussion with their group members. In the last 20 minutes of a lesson, the tutors' and students' questions were answered in a discussion summary. The lesson ended after the students' opinions about the teaching plan had been collected.

### Application of a 3C3R model in modifying a pediatric PBL teaching plan (The 9-step problem design process)

Based on traditional PBL courses, the 3C3R model was applied in order to analyze and evaluate the PBL teaching plan, involving the content, context, and connection among several parts of researching, reasoning, reflecting processes, and learning difficulties.

Content: Well-designed PBL problems should consider several aspects of the content components. First, problems must be aligned with curricular standards so that students' problem-solving and self-directed learning skill training and their domain/content knowledge proficiency will meet the standards. Moreover, teachers should use these standards to identify the major concepts and areas of the topic or subject, and then design PBL problems accordingly. Second, scope must be considered to ensure that the problems have sufficient breadth and depth. Achieving the proper scope can be accomplished by conducting content/task analyses of the learning goal. In the limited time frame of our PBL course, we sought to balance factual knowledge acquisition with development of problem-solving skills, including the alignment with the university curricular standards and ensuring the students' domain/content knowledge proficiency. For example, we only provided information that would typically be available to a physician in a clinical situation such as medical history, laboratory examinations and image materials. The information may however overstrain some students, not familiar with this new method of teaching. As long as the information and materials provided are complete and intelligible, the potential problem of students being overstrained should be avoided. The question, “is the information provided by medical history, laboratory examinations, and image materials complete and intelligible” therefore addresses this problem (question 7 as well) (Table S1). To further develop our 3C3R-PBL teaching plan, we integrated our experiences and varied the problem design or the information provided based on our results as well as coordinated our teaching particularly regarding domain knowledge.

Context: According to Hung, the context should lead to a contextual knowledge. He further emphasized that it is the authentic context of a problem that should motivate students to learn and will determine how actively the learners participate in the problem solving process. Authenticity includes contextual validity (problems should be relevant for the students' later work), degree of contextualization (the problem should be tailored so it is not too specific or too broad), as well as context relevance and proximity, which influences the degree to which the learners take ownership of the problems (students' intrinsic motivation). If the contextual components are poorly designed then student motivation to develop a self-directed problem-solving concept might be lowered [11]. The answer to the question “is the case context attractive” reflects mainly the interest of the students, thereby covering the above-mentioned items in one simple question (Table S1).

Connection: Students should be able to master the knowledge they learn in the lesson, which can be determined by their questions and the group discussion according to the connection of concept, principles and program of the medical knowledge. In our study connection included application of information about cyanosis, murmur, ischemia attack and others; the aims of the study included knowledge about cardiac embryonic developmental disorders, congenital cardiac malformations, fetal blood circulation characteristics, formation of post-natal hemodynamic changes, congenital heart disease classification and hemodynamic changes as well as congenital heart disease complications, prognosis and treatment principles, particularly the treatment of acute tet spells in children. The problem should be solved by using cyanosis differential diagnosis knowledge, and general differential diagnosis considerations of congenital heart diseases. Connection was evaluated with the question “can I discuss and connect disease related knowledge contents? (Table S1).

Researching: Hung stated in his 3C3R model, that goal design and context specifications should lead to an effective researching component of a problem. Defining specific problem goals should direct the systemic research and data collection processes, while context specification determines the initial frame of reference and influences what the students' choose to research and how they process the information related to the problem-solving tasks. He also emphasized that the learning should follow the conventional researching processes practiced by the professionals in the particular field [11]. In our study design, research pertained mainly to clinical practice conditions and the research process was guided by tutor observations of the students. For example, tutors assessed whether students were clearly aware of the current state of the problem, their state of known information (such as cyanosis, heart murmur) and unknown information, but the students had to establish their own knowledge base. The act of researching encompasses a variety of channels to collect information and to determine the reliability of data, and includes self-directed learning skills and the ability to analyze and summarize data by processing this information. The research design component of the model thus evaluated whether the students have exploration desire and whether they clearly understood the focus of the domain knowledge, as well as identified what further exploration was needed.

Reasoning: This learning component was related to how students analyzed the information pertinent to the problem. Questions included for example, what kinds of information are important? How to differentiate between central and peripheral cyanosis? What kind of diseases may result in cyanosis of children? What additional kinds of information are necessary from the patient to make further judgments? What is the significance for the patients of further laboratory examinations and other checks? How to decide a treatment for children? Thus the aim of the reasoning design was to let students learn how to accurately reason, how to analyze reasons when the group disagreed and to study whether they participated in a positive manner until the dispute was settled.

Reflection: In the case-based reflection process, questions assessed the student level of domain knowledge about congenital heart disease. For example, was the depth of learning appropriate? During PBL learning, did they use effective, efficient research methods? Did Tetralogy of Fallot tet spell lead to reasonable, effective reasoning processes? Were the students able to integrate their conceptual knowledge of congenital heart diseases? Did they develop effective problem-solving strategies? Thus, reflection helped the students achieve the best learning effect and let them master the knowledge after reflection and learning strategy adjustments.

The teaching plan based on CHD case studies and problems were designed following the 9-step problem design process.

1. Set goals and objectives
  Teaching goals: Students will master the clinical features of CHD. Teaching objectives: Students will gain experience in CHD diagnosis, becoming familiar with the clinical features, complications, as well as the prognosis and treatment of CHDs (atrial septal defect, ventricular septal defect, patent ductus arteriosus, tetralogy of Fallot).
  Objectives of domain knowledge: 1) Basic medicine: a) characteristics and disorders during fetal heart development as well as congenital heart malformation; b) characteristics of fetal blood circulation and changes of hemodynamics after birth; c) pathophysiology and anatomical characteristics of common CHD; d) mechanism of tet spells in pathophysiology and drug therapy; e) relationship between congenital heart malformation and genetic & environmental factors. 2) Clinical medicine: a) classification and b) ideas of diagnosis and therapeutic principles of CHD; c) clinical features and blood dynamic features common to CHD; d) auxiliary examinations of CHD (x-ray, ECG, echocardiogram, cardiac catheterization, and cardioangiography); e) complications, prognosis and treatment principles of CHD; f) therapeutic principles for tet spells.
  Objectives of skills for solving problem: 1) Master the pathophysiology, clinical features, auxiliary examinations, as well as complications of CHD 2) recognize the therapeutic principles of atrial septal defect, ventricular septal defect, patent ductus arteriosus, and tetralogy of Fallot; 3) recognize the pathogenesis and classification of CHDs; 4) recognize the characteristics of fetal blood circulation and changes of hemodynamics after birth; 5) understand the pathophysiology and anatomical characteristics of the pediatric circulation system.
  Objectives of self-directed learning ability: To master the pathoanatomy, hemodynamic changes, methods for diagnosis and differential diagnosis as well as treatment of CHDs.
2. Conduct content/task analysis)
  To analyze the content and task of the teaching plan “why the lips turn purple when a baby is crying” and to master the knowledge of CHD through the corresponding course.
3. Analyze context specification
  The designed content was determined by the factors that affect the researching and reasoning processes. In this teaching plan, the baby was a 4-month-old boy, whose lips turned purple when he cried. One morning, when he woke up and cried, his face suddenly became purple and he developed a tachypnea. Based on the patient's condition, the clinical features, diagnosis, complications, therapeutic principles and prognosis of a CHD were discussed.
4. Select/generate a PBL problem
  Possible problems included: 1) When a baby becomes cyanotic, what examinations should be done and what could be the relevant diseases? 2) Considering that systemic medical examination and cardiac auscultation were done and the clinical signs of the baby are available, what other examinations should be done next and how to diagnose and differentially diagnose an assumed disease? 3) How to treat the patient once the diagnosis was made? 4) What is the relationship between CHD and the characteristics of a fetal heart development?
5. Conduct PBL problem affordance analysis
  1. Problem situation: A 4-month-old cyanotic baby with tachypnea, who was sent to the pediatric hospital emergency room by his parents.
  2. Problem solving process: The baby was brought to the hospital by his parents, because they wanted to avoid “Blausucht” death. The diagnosis should therefore be based on the symptom of cyanosis. Thus, the problems were: What mechanisms are causing cyanosis? How to differentially diagnose central cyanosis and peripheral cyanosis? What is the common etiology of cyanosis? What clinical features and medical history is special for cyanosis? Following a systemic medical examination of the baby a heart murmur was observed by cardiac auscultation and a preliminary diagnosis of CHD was made. According to the medical history combined with the medical examination and auxiliary examinations, the next problem is to specify which kind of CHD the baby is suffering. Considering the features of the patient's medical history (tachypnea, face becomes purple when he was crying in the morning after he woke up) and based on his physical signs, it should be explained why cyanosis occurs in the baby and the relationship between cyanosis and CHD should be studied. Subsequently what kind of CHD could cause the cyanosis should be answered. What are the emergency treatment plans for a baby with cyanosis and tachypnea in the emergency room? How to treat the baby there after? What is the prognosis? What aspects should be taken into consideration for medical humanity?
  3. Domain knowledge needed to solve the problems.
  Concepts used in solving the problem: a) definition of cyanosis, concepts of central and peripheral cyanosis; b) concept of murmur, differential definition of functional and organic murmur; c) concept of CHD; d) pathoanatomy of common CHDs, concept of atrial septal defect, ventricular septal defect, patent ductus arteriosus, and tetralogy of Fallot; e) hemodynamic changes of CHD; f) concept of tet spells.
  Principles used in solving the problem: a) classification of CHD; b) clinical features of CHD; c) complications, treatment principles and prognosis of CHD; d) emergency treatment principles for tet spells; e) disorders during fetal heart development and congenital heart malformation; f) characteristics of fetal blood circulation and changes of hemodynamics after birth.
  Ideas for diagnosis and procedures for solving the problem: a) idea of cyanosis diagnosis; b) idea for CHD differential diagnosis.
6. Conduct correspondence analysis
  Context analysis of the problems are shown in Table S2.
  The context of the problem in which an anaerobic attack is a common phenomenon for tetralogy of Fallot, was appropriate for the students' study of CHD. In addition, the researching process of finding the related materials through the Internet to solve the problem was attractive for the students. Therefore, it satisfied the motivation of PBL problem design.
  Conclusions of correspondence analysis.
  Domain knowledge: 1) Correspondence between the expected target and range of PBL problems incorporates both basic medicine and clinical medicine. 2) Non-corresponding parts: a) the core domain knowledge in PBL problem design might not cover the following targets: “atrial septal defect, ventricular septal defect, patent ductus arteriosus”, “characteristics of fetal blood circulation and changes of hemodynamics after birth”. Therefore, in the problem design, discussions about what other CHDs might cause cyanosis should be introduced. Hence, the ideas for CHD diagnosis, classification for common CHDs, their clinical features and hemodynamic changes could be put forward. This is necessary for the diagnosis, reasoning, and differential diagnosis of CHD. b) Some peripheral knowledge may be beyond the target range, for example, incidence of CHD, relationship between congenital heart malformation and genetic & environmental factors.
  Context information: the context is well established based on the context information of the problems.
  Problem solving skills and self-directed learning abilities: 1) Research part: Students need to research and compare some information such as medical history (cyanosis, tachypnea) and physical examination (cardiac murmur). This information should be collected to complete the diagnosis, and a preliminary hint should be provided if necessary in the problem design to guide the students finding the information. 2) Reasoning part: The students should identify the cause of cyanosis, analyze the functional and organic murmur, and specify what concepts, principles and procedures are needed for correct CHD reasoning (Table S2).
7. Conduct calibration processes:
  For students, how to diagnose the baby's disease? The baby's clinical feature is cyanosis, which can be induced by circulatory system diseases, respiratory diseases or hematological disease. Combined with a medical examination and the finding that the baby has cardiac souffle, what might be the most likely disease? What auxiliary examinations should be done further? What is the significance of these examinations? For the case that the baby suffers from CHD, how to differential diagnose CHD and what are the clinical features of common CHD (ASD/VSD/PDA/TOF)? What is different in pathological hemodynamic changes for different CHDs? What is the treatment and prognosis? What details should be noticed in the doctor-patient communication of diagnosis and treatment?
8. Construct reflection component (The same problem expression as in calibration processes):
  Discussions were conducted in subgroups and the researching and learning process was based on PBL. Students' tasks were to collect and organize information from relevant web pages and sources, to confirm the symptoms and examinations, to summarize features of the medical history and to draw a conclusion, i.e. to identify the symptoms and analyze the problem, to determine the cause of cyanosis, and to conduct researching, reasoning, and reflecting process in the discussion. Each group held 2 group conferences after a given lesson, which fostered cooperative learning activities in various forms such as discussion, consultation and debate. In the conference, each member reported the information he/she had found and presented his or her personal point-of-view. The conference discussions were recorded, and a plan for further work was completed. Eventually, a conclusion of a diagnosis was drawn for the baby and a treatment plan developed to be carried out. Two weeks later, the results of each group were presented in a class meeting and the group provided a report in the form of a word file, PPT, webpage, or other form.
9. Examine inter-supporting relationships of 3C3R components
  The correspondence analysis between cognitive process of problem solving and learning targets was examined as shown in Table S3.

### Effectiveness evaluation method

#### Questionnaire survey

After the PBL summary lesson, the PBL problem design of the teaching plans were evaluated by questionnaire surveys of students and tutors, with participation rates of 100%. Surveys included the following 7 aspects: Content: Was the information provided by medical history, laboratory examinations and image materials complete and intelligible? Context: Was the case context attractive. Connection: Can you discuss and connect heart disease related knowledge? Researching: Were you willing to undertake your own research in the learning processes? Reasoning: Did you have proper reasoning through discussion? Reflecting process: Was there a reflection process in the learning process? In addition, a question about the learning difficulty in this teaching plan was added. Scores: 1, strongly disagree; 2, disagree; 3, not sure; 4, agree; 5, strongly agree (Table S1).

### Statistics

Data were analyzed with the SPSS version 13.0 software. Quantitative data are expressed as mean±SD, a Chi-square test or Fisher`s exact test was applied for comparison of the PBL and 3C3R-PBL groups. For the Questionnaire scoring of students and tutors, comparative analysis of PBL and 3C3R-PBL groups was done using Two-way ANOVA, and the examination score of students between the two groups was analyzed with a Nonparametric test, p-values of less than 0.05 were considered statistically significant.

## Results

### Differences between PBL and 3C3R-PBL in correspondence analysis of problem solving cognitive processes and learning targets

Mutually supportive relationships between teaching plan components were studied (Table S4), including the correspondence analysis of contents and problem solving cognitive processes and learning objectives. In 3C3R-PBL the conclusions in diagnosis based on auxiliary examinations, such as the chest radiography, ECG and cardiac color Doppler ultrasound were omitted. In addition, in the traditional PBL, there was little discussion about the relationship between the occurrence of congenital heart malformations and genetic & environmental factors, the incidence of CHD in China, and how to protect pregnant women more effectively from the occurrence of fetal CHD, which were included in the 3C3R-PBL course.

### Questionnaire survey results

#### Questionnaire survey results of traditional PBL and 3C3R-PBL students

The proportion of students in the traditional PBL course that scored ≥4 for content was 90.8% suggesting that the information on medical history, laboratory examinations and image materials was believed to be complete and intelligible. For context, 80.3% of students in the traditional PBL course scored ≥4, indicating that they believe that the context was attractive. However, only 64.5% of the students scored discussion as ≥4 demonstrating that they felt they could discuss and connect heart disease related knowledge and 5.3% felt unable to find any emphasis on the symptom connections. Of all students in the traditional PBL course 81.6% were willing to engage in research during the learning processes; 55.3% used proper reasoning through discussion, while 5.3% found mistakes in their reasoning process; 40.8% believed that there was a reflection process, while 2.6% considered the reflection process insufficient. Lastly, only 31.6% of the students believed that the teaching plan was difficult and scored ≥ 4 (Table 2).

**Table 2:** Comparison of traditional PBL and 3C3R-PBL questionnaire survey results from students and tutors.

Item	Score*	Number of Student (%)		p-value	Number of Tutor (%)		p-value
		PBL	3C3R-PBL		PBL	3C3R-PBL	
		76	63		7	6	
Content	≥4	69(90.8)	63(100.0)	0.7159	7(100.0)	6(100.0)	1.0000
Context	≥4	61(80.3)	62(98.4)	0.4577	5(71.4)	6(100.0)	1.0000
Connection	≥4	49(64.5)	57(90.5)	0.1899	2(28.6)	5(83.3)	0.3742
Researching	≥4	62(81.6)	60(95.2)	0.5375	5(71.4)	6(100.0)	1.0000
Reasoning	≥4	42(55.3)	56(88.9)	0.0731	7(100)	6(100.0)	1.0000
Reflecting	≥4	31(40.8)	48(76.2)	0.0346^★^	4(57.1)	6(100.0)	0.6802
Difficult	≥4	24(31.6)	58(92.1)	0.0003^★★★^	3(42.9)	6(100.0)	0.4149

Note: * Scores: 1, strongly disagree; 2, disagree; 3, not sure; 4, agree; 5, strongly agree.

★,★★★ The significance difference of test scores between 3C3R-PBL and PBL, was defined as P<0.05 (^★^) and P<0.001 (^★★★^).

All students who participated in a 3C3R-PBL problem designed course believed that the medical history, laboratory examinations and images materials were complete and intelligible; (score ≥4 100%). For context, 98.4% of students scored ≥4, showing that the context was attractive. In the discussion, 90.5% of the students indicated that they could draw connections among the disease symptoms. Most of the students, 95.2% were willing to conduct research during the learning processes; 88.9% used proper reasoning through discussion, and no student found a mistake in their reasoning process; 76.2% believed that there was a process of reflection, while 3.2% considered the reflection processes lacking. Lastly, 92.1% believed that the teaching plan was difficult/challenging and scored ≥ 4 while only 3.2% thought that the plan was easy (Table 2).

#### Questionnaire survey results of traditional PBL and 3C3R-PBL tutors

All tutors in the traditional PBL groups believed that the information on medical history, laboratory examinations and image materials were complete and intelligible and 71.4% believed that the context was attractive, scoring ≥4. Only 28.6% believed that the students' discussion was based on the connections among symptoms; 71.4% believed that the students were willing to conduct research in the learning processes and scored ≥4 and all tutors agreed that the students used proper reasoning; 57.1% thought there was a reflection process during the learning; 42.9% believed that the teaching plan was difficult and scored ≥4 (Table 2). All tutors in 3C3R-PBL groups believed that the information on medical history, laboratory examinations and image materials were complete and intelligible and all believed that the context was attractive; of all 3C3R-PBL tutors 83.3% believed that the students' discussion was based on the connection among symptoms and all believed that the students were willing to conduct research during the learning processes; all agreed that the students used proper reasoning; they all believed that there was a reflection process in the learning; 42.9% believed the teaching plan was difficult and scored ≥4, while 66.7% scored difficult as 5 (Table 2).

#### Score comparison of the questionnaire surveys between PBL and 3C3R-PBL groups of students and tutors

**Figure 2:**
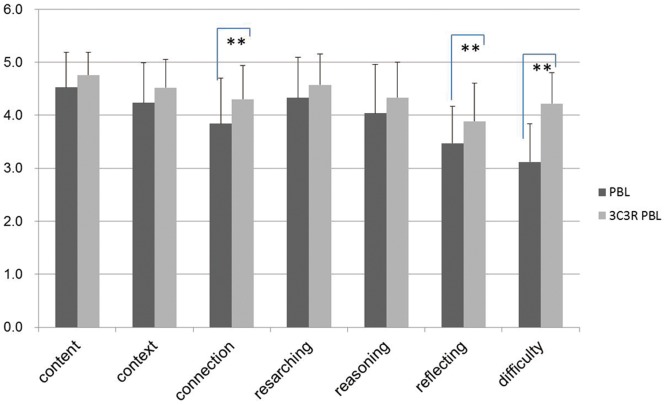
Comparison of test scores from students for 3C3R-PBL and PBL.

**Figure 3:**
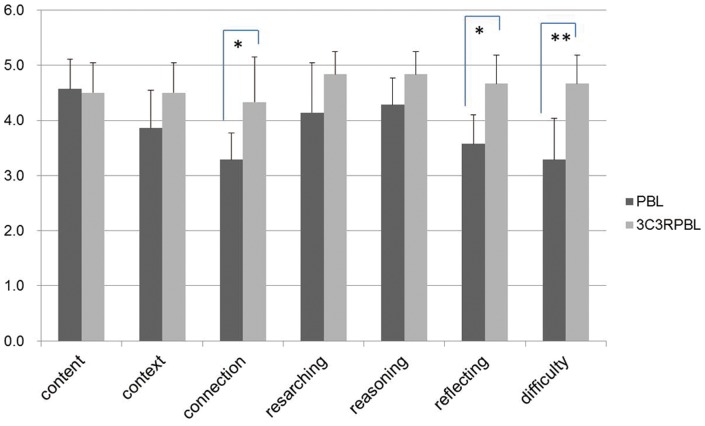
Comparison of test scores from tutors for 3C3R-PBL and PBL.

**Figure 4:**
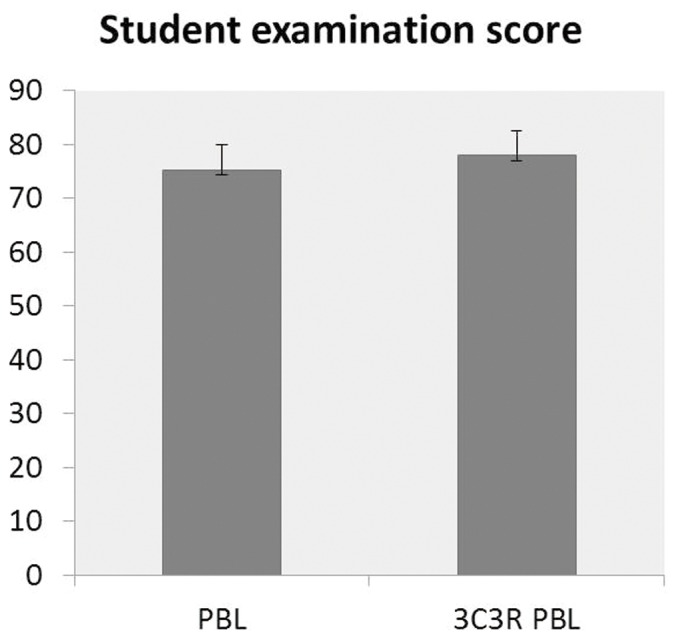
Comparison of overall test scores from students for 3C3R-PBL and PBL.

The students and tutors were convinced by the content, case contexts, research process and reasoning process of the teaching plan. Students' scoring of connection and reflecting were significantly improved when the PBL plan was amended by a 3C3R model, p<0.01 and the learning difficulty opinion of the case was statistically increased, p<0.01 indicating that they found the 3C3R model more challenging (Figure 2). Similarly for tutors' the connection and reflecting scores were significantly improved, p<0.05; also the opinion about the case learning difficulty was statistically increased, p<0.01 (Figure 3). The test scores of the students for PBL and 3C3R-PBL teaching showed that they considered the 3C3R-PBL teaching method to be significantly better than the PBL method (Figure 4).

## Discussion

In this study, we demonstrated that students participating in learning processes with a “real” story learn more in depth via their complex task and perform better. Students' critical thinking abilities are improving, they become more effective readers and writers and are thus provided with enhanced skills to solve complex problems rather than merely memorizing facts or developing step-by-step processes for learning through restrictive tasks or the application of simple assessments [17], [18]. PBL problems should therefore come from real-life pediatric science, making the contextualized learning of knowledge or skills achievable, rather than from book materials with high-level structured and contextualized knowledge over view. The core components of a 3C3R problem design model are content, context and connection. Content is the first core component of 3C3R problem design model. Previous works have indicated that students taking PBL courses generally perform a little worse in the test of contents than those taking traditional courses [19]. Researchers attributed this to the fact that PBL may miss the attention to learn content breadth and factual knowledge acquisition due to its emphasis in content depth and higher-order thinking [20]. For example, in this study, during discussion of the condition of children with CHD, the professional knowledge needed to be learned and mastered prior to group discussion, otherwise PBL learning lacks the necessary content. Therefore, a 3C3R concept is a necessary complement of the PBL teaching mode. The second core component of a 3C3R problem design model is context. In order to become effective problem solvers in a particular area, students need to gain not only sufficient domain knowledge but also specific situational knowledge, which is implicit but essential to effectively solve problems [21], [22]. The third core component of a 3C3R problem design model is connection. In general, in a PBL course, students organize their knowledge structure based on problems. PBL courses often contain a series of questions, which cover all aspects of the course. By using a case study, the knowledge can be “packed” into a problem or issue collection, so that students can effectively obtain relevant knowledge, which they will then be able to effectively recall and add to related knowledge, should they encounter the same or similar questions in practice.

However, effective problems do not necessarily guarantee that the desired satisfactory learning outcome will be achieved. Studies have previously shown that learning outcomes may be restricted by the lack of guidance and promotion in the learning process [23], [24]. In this study, we attempted to ensure that the desired learning outcomes of the PBL course were achieved by applying the process components of the 3C3R problem design model, whose aims promote and support students' thinking and participation. The process components include researching, reasoning and reflecting, which are dynamic elements related with the static core elements in the 3C3R model. The main functions of these dynamic elements are: 1) to guide the students' efforts toward the expected learning goals; 2) to adjust the required cognitive processing level according to the student's preparation level and cognitive state; and 3) to reduce students' discomfort when they join the PBL course for the first time [12]. Therefore, the general objective of 3R is to promote meaningful participation in problem-solving processes, and to train the students to be effective and efficient learners and problem solvers.

The results of this study indicated that the content, case context, research process, and reasoning process of the PBL teaching plan, after revision using a 3C3R model, were convincing to the students and tutors; scores of connection, and reflecting were significantly improved, and case learning difficulty was increased. The process of researching the case provides for mutual support among content, context, connection and researching, taking into concern the context and inner connection of the plan, the coherent echo of contents, and the clear, specific and complete statement of a problem [25]. The designed problems should be hierarchical, systematic and integrated thereby allowing students to adopt a correct and effective research method, leading them to analyze and choose the right strategy for solving problems, supporting their abilities to gain knowledge in the content, guiding them during researching and in organically integrating the gained conceptual knowledge into previously obtained knowledge. Moreover, if the learning difficulty of the teaching plan can be increased, it will be more challenging and will therefore increase students' interests in the study.

The reasoning component of the teaching plan should be fully supported by the context design as well as by connections among problems within the case study to allow the knowledge gained to be integrated into previously obtained knowledge [5]. Diagnostic conclusions based solely on auxiliary examinations, such as chest images, ECG and cardiac color Doppler ultrasound; however, they do not lead to the development of a deeper knowledge because the reasoning process is lacking. In the 3C3R designed teaching plan, the conclusions in diagnosis based on auxiliary examinations, such as the chest images, ECG and cardiac color Doppler ultrasound, were omitted. Only picture information was available so students used their own logical reasoning processes to form their conclusions.

Our survey results indicated that some students thought the learning plan was moderately difficult, while a few students thought the teaching plan was too simple, and the tetralogy of Fallot diagnosis was too easy. The problem design failed to motivate these students to seek out addition information and therefore did not train them in active exploration of problems. Reflecting is not supported by acquiring content knowledge, it must instead be drawn from conclusions arrived through analysis. The traditional PBL teaching plan did not support reflection, which affected the systematic and conceptualization integration of the related CHD knowledge. In the 3C3R modified PBL course version, the information of conclusions in diagnosis based on auxiliary examinations was omitted, which increased the difficulty in correct diagnosis of the case, and as a result, the learning plan became more challenging. The feedback of students and tutors indicated that the reflecting process was improved.

Limitations of our study included its small sample size of only 139 fifth grade students of eight-year studying courses in the medical school of Shanghai Jiaotong University and only 13 tutors participating in the PBL lessons. Further, the teaching contents were focused only on pediatric congenital heart diseases. To gain more information on the general acceptance of 3C3R-PBL courses, a larger number of students should be evaluated and the teaching topics should be expanded to include a variety of diseases, such as general newborn diseases, pediatric respiratory diseases, pediatric neurological diseases as well pediatric digestion system diseases, with a more complex problem design.

We believe that this was an important study in that it was the first time that the 3C3R model was introduced in a pediatric PBL lesson “why the lips turn purple when a baby is crying” and therefore benefited the PBL lesson designer to systematically design an effective problem and course in a clinical teaching program, which is in contrast to other medical teaching in China highly related to the daily clinical practice, while designed by practicing physicians. Taken together a 3C3R model is helpful in PBL problem design of Pediatrics teaching and enhances problem-based learning, improves medical students' ability to analyze and solve problems, and trains them in their self-oriented learning ability. The significant positive improvement during exploring an establishment of the 3C3R model for a pediatric teaching PBL problem design might be a benefit for training pediatric medical personnel as well as for an effective promotion and development of a general PBL teaching concept in China.

## Supporting Information

Table S1 **Questionnaire survey form.** Notes: 1.Completely disagree; 2. Disagree; 3. Neutral; 4. Agree; 5. Completely agree.
(TIF)

Table S2 **Correspondence Analysis of Content.** Notes: 1. Pathogenesis; 2.Pathoanatomy; 3. Pathophysiology; 4. Clinical feature; 5. Auxiliary examination; 6. Treatment and prognosis; 7. Cyanosis identification; 8. CHD identification; 9. Precondition; 10. Beyond target range.
(TIF)

Table S3 **Correspondence analysis between cognitive process of problem solving and learning targets.** Notes: 1. Confirming and collecting all researching information needed with aid; 2. Simple reasoning with aid; 3. Choose the most practicable plan for diagnosis and treatment.
(TIF)

Table S4 **The mutual support relations between 3C3R components.**
(TIF)
