# Supplementary figures and images for: 3C3R Modified PBL Pediatric Teaching of Chinese Medical Students
Source: PLoS One. 2013 May 7;8(5):e63412. doi: 10.1371/journal.pone.0063412 (PMC3646730; doi:10.1371/journal.pone.0063412)

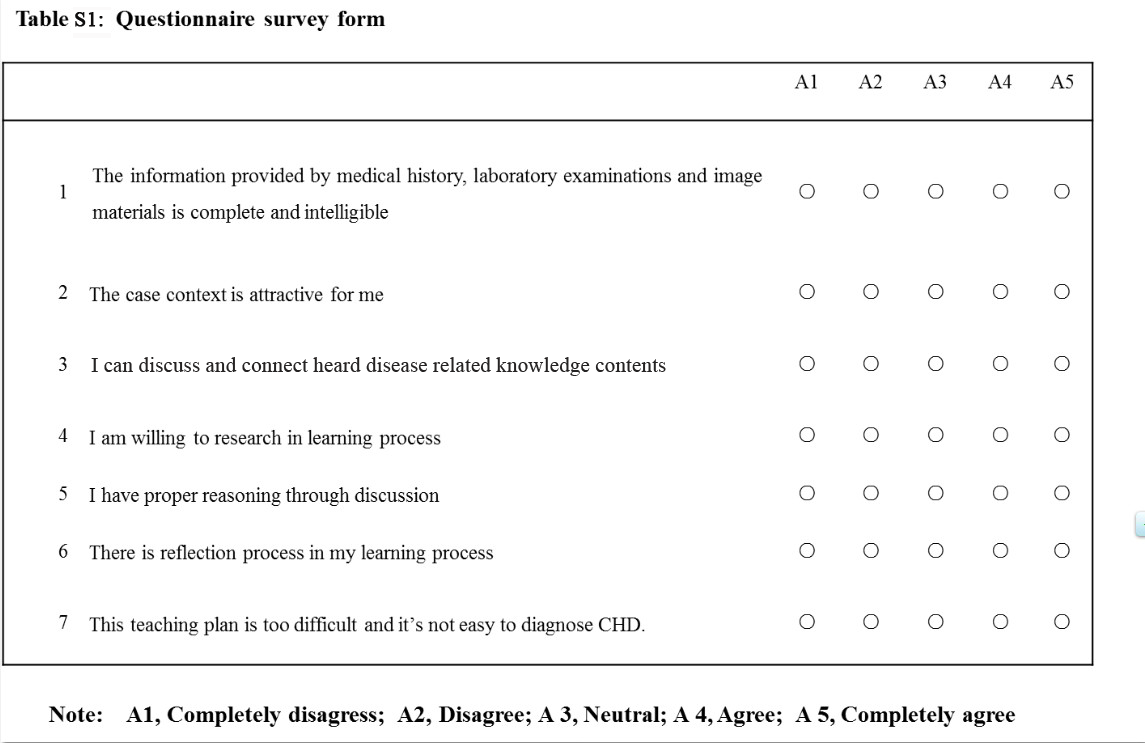

Supplement: Table S1 — Questionnaire survey form. Notes: 1.Completely disagree; 2. Disagree; 3. Neutral; 4. Agree; 5. Completely agree. (TIF) [file pone.0063412.s001.tif]

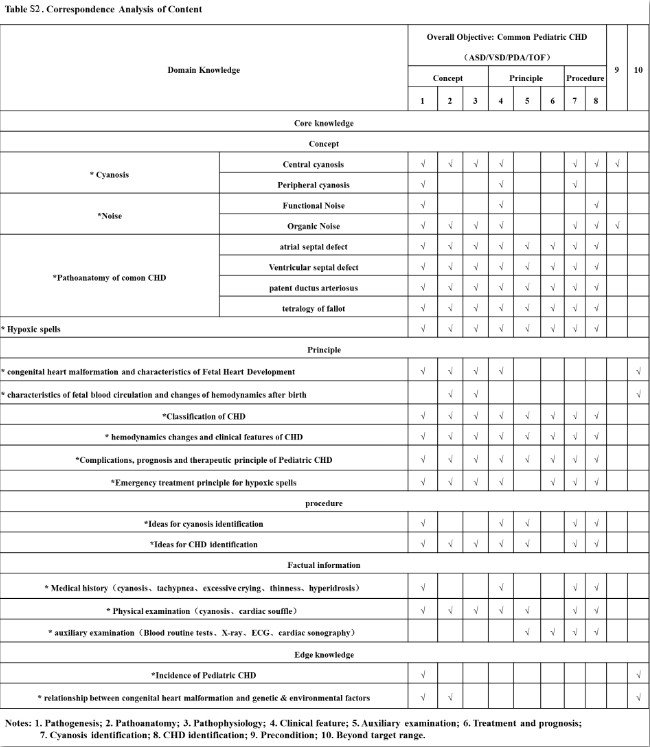

Supplement: Table S2 — Correspondence Analysis of Content. Notes: 1. Pathogenesis; 2.Pathoanatomy; 3. Pathophysiology; 4. Clinical feature; 5. Auxiliary examination; 6. Treatment and prognosis; 7. Cyanosis identification; 8. CHD identification; 9. Precondition; 10. Beyond target range. (TIF) [file pone.0063412.s002.tif]

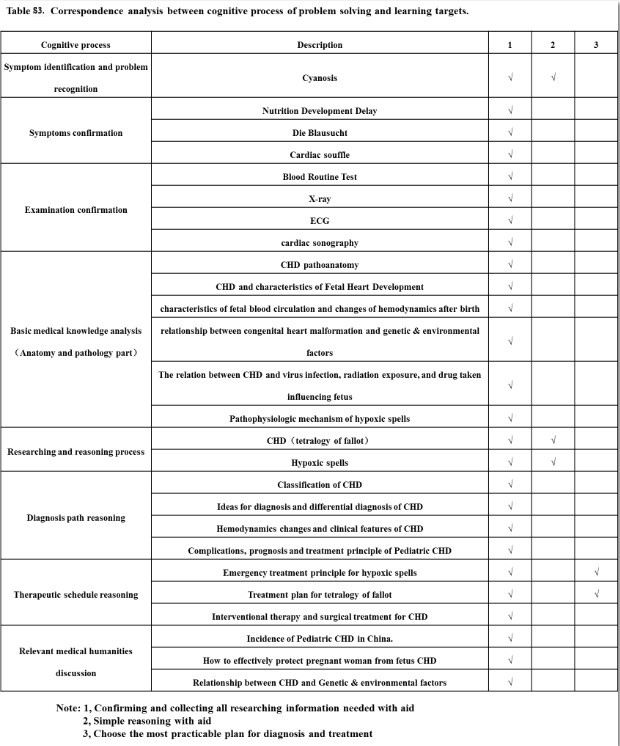

Supplement: Table S3 — Correspondence analysis between cognitive process of problem solving and learning targets. Notes: 1. Confirming and collecting all researching information needed with aid; 2. Simple reasoning with aid; 3. Choose the most practicable plan for diagnosis and treatment. (TIF) [file pone.0063412.s003.tif]

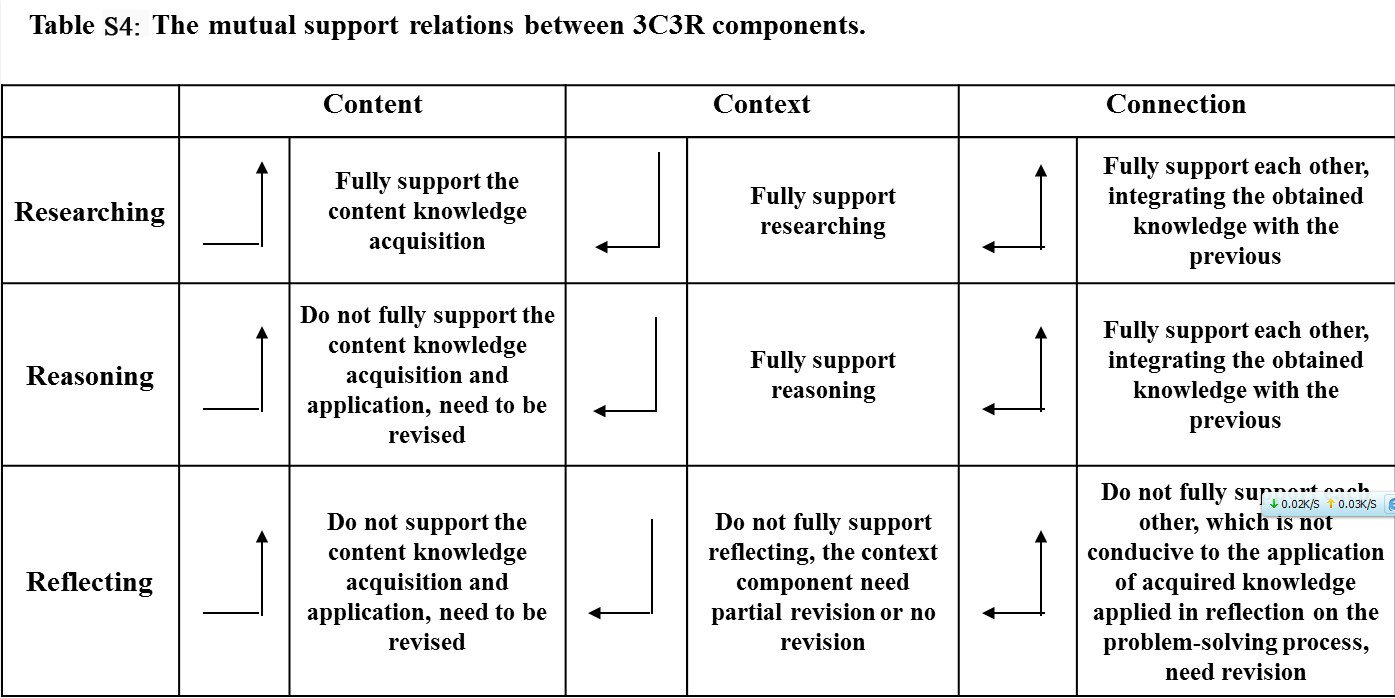

Supplement: Table S4 — The mutual support relations between 3C3R components. (TIF) [file pone.0063412.s004.tif]
